# Chromatin signaling in muscle stem cells: interpreting the regenerative microenvironment

**DOI:** 10.3389/fnagi.2015.00036

**Published:** 2015-04-07

**Authors:** Arianna Brancaccio, Daniela Palacios

**Affiliations:** Laboratory of Epigenetics and Signaling, IRCCS Fondazione Santa LuciaRome, Italy

**Keywords:** muscle regeneration, satellite cells, skeletal muscle, epigenetics, signaling pathways, sarcopenia

## Abstract

Muscle regeneration in the adult occurs in response to damage at expenses of a population of adult stem cells, the satellite cells. Upon injury, either physical or genetic, signals released within the satellite cell niche lead to the commitment, expansion and differentiation of the pool of muscle progenitors to repair damaged muscle. To achieve this goal satellite cells undergo a dramatic transcriptional reprogramming to coordinately activate and repress specific subset of genes. Although the epigenetics of muscle regeneration has been extensively discussed, less emphasis has been put on how extra-cellular cues are translated into the specific chromatin reorganization necessary for progression through the myogenic program. In this review we will focus on how satellite cells sense the regenerative microenvironment in physiological and pathological circumstances, paying particular attention to the mechanism through which the external stimuli are transduced to the nucleus to modulate chromatin structure and gene expression. We will discuss the pathways involved and how alterations in this chromatin signaling may contribute to satellite cells dysfunction during aging and disease.

## Muscle Regeneration

Adult muscle is a very stable tissue, with very few fibers being replaced during the normal life of the organism. However, it has a remarkable capacity to regenerate in response to tissue damage. Upon injury, either physical or genetic, changes in the injured microenvironment (i.e., necrosis of damaged fibers, recruitment of the inflammatory infiltrate and release of cytokines and growth factors) lead to the activation, expansion and differentiation of a population of muscle-resident stem cells called satellite cells (Chargé and Rudnicki, 2004). Satellite cells were originally named after their anatomical position beneath the basal lamina of muscle fibers and are characterized by the expression of the transcription factor Pax7 (Mauro, 1961; Cornelison and Wold, 1997; Seale et al., 2000; Chargé and Rudnicki, 2004). Upon activation, satellite cells start to proliferate and up-regulate the early Muscle Regulatory Factors (MRFs) Myf5 and MyoD. After several rounds of cell divisions cells down-regulate Pax7 and induce the expression of the late MRFs (Myogenin and MRF4) and of the cell cycle inhibitor p21. The differentiation program then culminates with the expression of structural and contractile proteins and the fusion of differentiating muscle cells to repair the damaged fibers. Not all activated satellite cells complete the differentiation program and after asymmetric division a small subset of Pax7-positive/MyoD-negative progenitors exit the cell cycle and re- enter quiescence to replenish the satellite cell pool (Chargé and Rudnicki, 2004; Wang et al., 2014).

To achieve their goal satellite cells need to undergo a complex remodeling of chromatin that temporally activates and represses discrete transcriptional programs. Such remodeling is determined by changes in the composition of the satellite cell niche that are transmitted to the nucleus through several cytoplasmic cascades. Here we will discuss how such cascades signal to the chromatin of satellite cells to define the transcriptional response that allows them to proceed through the myogenic program.

At the molecular level, the signaling pathways and transcription factors orchestrating muscle regeneration have been extensively studied. Briefly, myogenesis is controlled by the sequential action of lineage determination markers (i.e., Pax3/Pax7) and early and late MRFs, that act together with Mef2 and Six proteins to recruit chromatin modifying complexes and regulate muscle gene expression. Reprogramming of satellite cells nucleus entails the coordinated activation and repression of discrete subset of genes to progress through the myogenic program. For instance, committed myoblats keep myogenic identity but repress late muscle genes while continuing to proliferate. Finally, cells switch-off lineage-determination genes and genes necessary for cell cycle progression and activate late muscle-differentiation markers (reviewed in Palacios and Puri, 2006; Guasconi and Puri, 2009; Segalés et al., 2014). Recently, the introduction of high throughput genome-wide studies has been fundamental to provide a deeper insight into the transcriptional and epigenetic programs that modulates myogenesis. By combining ChIP-seq to gene expression analysis, the group of Tapscott showed that MyoD binds to thousands of non-canonical sites both in myoblasts and myotubes, which coincides with local histone hyperacetylation but not necessarily with gene activation. Upon induction of differentiation MyoD-binding to a preferred E-box motif (CAGGTG) located within an accessible chromatin context leads to muscle-specific gene expression (Cao et al., 2010; Fong et al., 2012). Therefore, both genetic (the specific E-box sequence) and epigenetic (local chromatin structure) determinants are fundamental for the activation of the correct differentiation program. Recently, Asp et al. provided an exhaustive analysis of the differentiation-associated epigenetic changes in muscle cells (Asp et al., 2011) and identified novel muscle-specific enhancers associated to MyoD binding (Blum et al., 2012; Blum and Dynlacht, 2013). To add complexity to this scenario, the lab of Sartorelli showed that some muscle-specific enhancers are actually transcribed in what have been called enhancer RNAs (eRNAs), which function as novel epigenetic regulators of gene expression (Mousavi et al., 2013). It is therefore becoming increasingly clear that modulating the epigenome is an essential mechanism that allows muscle cells to proceed through the differentiation program.

Before getting in depth into the mechanisms by which signaling molecules act to modulate the epigenome of satellite cells, it is important to highlight some of the key aspect of epigenetics and its role in regulating gene expression during cellular differentiation.

## Epigenetics: From the DNA Sequence to Differential Gene Expression

Historically introduced by Conrad Waddington in 1942, the term epigenetics was coined to explain why specialized cells in a multi-cellular organism exert different functions despite having the same genetic material. This is accomplished by the addition of epigenetic modifications, covalent modifications of the DNA and associated proteins that determine the activation and repression of discrete transcriptional programs during development, cellular differentiation and disease (reviewed in Bernstein et al., 2007; Bergman and Cedar, 2013).

The first layer of epigenetic regulation is the covalent modification of DNA. Mammalian genomes are globally methylated (Eckhardt et al., 2006; Bergman and Cedar, 2013). In eukaryotes, the majority of DNA methylation occurs on cytosines that precede a guanine nucleotide (CpGs). CpG dinucleotides are not evenly distributed through the genome. Ninety eight percent of the dinucleotides are located in regions of low-CpG density, are usually methylated through the genome and may be demethylated in a tissue-specific fashion (Bergman and Cedar, 2013). The remaining two percent of CpGs cluster in regions termed CpG islands (Bird et al., 1985; Gardiner-Garden and Frommer, 1987), stretches of 500–1000 base pairs of DNA that have a higher CpG density than the rest of the genome and are usually kept free of methylation in all tissues (Bergman and Cedar, 2013). Unmethylated CpG islands are associated to DNA sequences more accessible to transcription factor binding, resulting in enhancement of gene expression (Tazi and Bird, 1990; Ramirez-Carrozzi et al., 2009; Choi, 2010). On the contrary, when CpG islands are methylated gene expression under their control is silenced. This is achieved through the binding of Methyl-CpG-binding proteins (MBD; Nan et al., 1993; Hendrich and Bird, 1998; Hendrich et al., 2001), which recognize 5-methyl cytosine (5mC) and recruit repressor complexes leading to changes in chromatin structure (Nan et al., 1998; Ng et al., 1999; Wade, 2005). Aberrations in the DNA methylation pattern are a common feature of many cancers and have been also observed in aging tissues (Feinberg and Tycko, 2004; Bergman and Cedar, 2013).

DNA methylation is catalyzed by DNA methyltransferases (Dnmts). Of them, Dnmt1 is responsible for maintaining methylation after DNA replication whereas Dnmt3a and Dnmt3b catalyze *de novo* DNA methylation (Goll and Bestor, 2005). It has been recently demonstrated that DNA methylation is reversible thanks to the action of the family of ten-eleven translocation methylcytosine dioxygenases (Tet). Tet proteins modify 5mC to generate 5-hydroxymethyl-cytosine (5hmC; Kohli and Zhang, 2013; Piccolo and Fisher, 2014). 5hmC can be then subjected to subsequent chemical modifications producing different intermediates that are recognized and cleaved off by TDG (thymine DNA glycosylase) and replaced with an unmethylated cytosine (He et al., 2011). The conversion of 5mC to 5hmC impairs the binding of the repressive methyl-binding protein such as MeCP2 and plays an important role in regulating gene expression (Valinluck et al., 2004).

The DNA methylation pattern influences and is also influenced by histone modifications, the second layer of epigenetic modifications. In eukaryotic cells the DNA is packed together with histones and other proteins to fit into the limited space of the nucleus, in a structure called chromatin. The basic unit of the chromatin is the nucleosome that is made up of 147 base pairs of DNA wrapped around an octamer of histone proteins called “core”, which contains two copies of each histone, (H2A, H2B, H3, H4) (Luger et al., 1997). Each histone has an N-terminal tail that protrudes from the nucleosome core and is target of different types of covalent modifications such as acetylation, methylation, o-glycosilation and ubiquitylation. Post-translational modifications of histones tails (and bodies) regulate chromatin structure and accessibility (Bannister and Kouzarides, 2011; Tessarz and Kouzarides, 2014). Acetylation of several lysine residues is usually associated with gene activation and is catalyzed by histone acetyltransferases (HATs) such as p300 and PCAF, while it is erased by histone deacetylases (HDACs; Kouzarides, 1999). Differently from histone acetylation, methylation occurs on both lysines and arginines and can either activate and repress gene expression (Ng et al., 2009). Amongst the histone methyltransferases (HMTs), particular interest has been put on the enzymes responsible for H3K27 and H3K9 di- and tri-methylation as these modifications are considered key regulators of gene repression. The first lysine methyltransferase identified was Suv39h1 (KMT1A) that targets H3K9. H3K9 me2/3 mark is recognized by HP1 proteins, which recruit Suv39h1 to the chromatin to spread the repressive mark (Lachner et al., 2001; Bannister and Kouzarides, 2011). Ezh2, the catalytic subunit of the Polycomb Repressive Complex 2 (PRC2), tri-methylates K27 on H3 to keep genes in a repressed state (Margueron and Reinberg, 2011; Riising et al., 2014). H3K27me3 creates a docking site for PRC1, which ubiquitylates H2A, leading to Dnmt recruitment and to a more compact chromatin structure (Sparmann and van Lohuizen, 2006). However, this view has been challenged recently and two studies have shown PRC1-mediated H2AK119ub is sufficient to trigger PRC2 recruitment (Blackledge et al., 2014; Cooper et al., 2014). Histone methylation is reversible and histone demethylases for most of the modified residues have been described (Klose and Zhang, 2007; Ng et al., 2009).

Based on the different combinations of modifications, chromatin can either activate (i.e., H3K4me3, H3K9Ac) or repress (H3K9me2/3, H3K27me3) gene expression. Further, in some cases it can prime associated genes for future regulation (Schneider et al., 2004; Azuara et al., 2006; Bannister and Kouzarides, 2011; Margueron and Reinberg, 2011). For example, in undifferentiated embryonic stem (ES) cells, developmental regulatory genes contain bivalent domains that are characterized by the co-existence of opposing modification in terms of activation (H3K4me3) and repression (H3K27me3). This allows keeping “marked” genes poised in a silenced state for being rapidly activated or repressed upon differentiation process (Azuara et al., 2006; Voigt et al., 2013).

DNA and histone modifications are therefore essential components of an epigenetic program that ultimately modulates chromatin accessibility to transcription factors and chromatin remodelers and needs to be tightly regulated. For instance, it has been previously shown that hyperacetylated chromatin is preferentially remodeled by chromatin remodeling complexes, whereas hypoacetylation leads to a more compact structure. Chromatin remodeling is accomplished by different families of proteins that use the energy derived from ATP to disrupt or modify nucleosome positioning (Saha et al., 2006). The best-studied chromatin remodelers are the SWI/SNF and ISWI complexes (Deuring et al., 2000; Martens and Winston, 2002). Both families contain a conserved ATPase subunit and a set of additional, conserved core members. SWI/SNF ATPases Brg1 and Brm contain one or multiple bromo-domains that bind acetylated histone tails (Winston and Allis, 1999; Kasten et al., 2004) while ISWI has two domains (SANT and SLIDE) that allow the recognition of the histone tails and the DNA linker (Grüne et al., 2003). In addition, SWI/SNF and ISWI complexes contain structural subunits that contribute to differential gene-specific targeting.

In summary, the high dynamicity and plasticity of the chromatin, which can go from permissive to refractory structures and *vice-versa*, is an important pre-requisite for the proper control of gene transcription. Here we will discuss how chromatin function is modulated during muscle regeneration in response to external cues to activate and repress discrete subset of genes.

## When Signaling Cascades get into the Nucleus

Satellite cells receive a myriad of signals from the regenerative microenvironment that guide the cells through the multi-step process leading to activation of a quiescent stem cells pool, expansion of the activated muscle progenitors and induction of the terminal differentiation program to induce regeneration of damaged muscles. Necrotic cues released from the damaged fibers, cytokines secreted by the inflammatory infiltrate, growth factors, free radicals, soluble proteins and changes in the extra-cellular matrix composition alter the satellite cell niche and need to be interpreted (Guasconi and Puri, 2009).

The precise composition of the extra-cellular environment is therefore fundamental to determine the gene expression profile that will guide the step-wise progression from quiescent satellite cells into multinucleated myofibers. Such information is transmitted to the cell nucleus through several cytoplasmic cascades that ultimately target components of the myogenic transcriptosome and chromatin modifying enzymes to regulate gene expression. Here we will review our current knowledge on how extra-cellular signals are transmitted to the chromatin of muscle cells to coordinately regulate the gene expression program needed for muscle regeneration. We will discuss the pathways and key signaling molecules involved as well as the downstream effectors and the changes in chromatin structure and function necessary to activate or repress discrete gene expression programs. The exquisite coordination of such programs may be subject to alterations by both cell-extrinsic changes (changes in the microenvironment) and cell-autonomous modifications (i.e., changes in the metabolome or in the epigenome). Understanding how this chromatin signaling modulates cell fate will assist us in the rational design of pharmacological therapies to improve satellite cell function in physiological (i.e., aging) or pathological (i.e., muscular diseases) conditions.

### Calcium-Dependent Chromatin Signaling

The importance of chromatin signaling in the control of muscle gene expression was first highlighted by work from Olson’s laboratory. In their pioneer work McKinsey et al. showed how calcium calmodulin kinase (CamK) activity is necessary to release class II HDACs from Mef2 transcription factors at the onset of muscle differentiation (McKinsey et al., 2000). Mef2 belong to the MCM1-Agamous-Deficiens-Serum response factor (MADS) box domain family of transcriptional regulators that cooperate with the MRFs (Myf5, MyoD, Myogenin and Mrf4) to activate muscle gene transcription (Puri and Sartorelli, 2000; Potthoff and Olson, 2007). In proliferating myoblasts Mef2 binds to and recruits class II HDACs (HDAC4 and 5) to the chromatin of muscle genes, acting as a transcriptional repressor (Lu et al., 2000). Upon induction of differentiation, activation of CamK in response, at least in part, to Insulin Growth Factor I (IGF-1), leads to phosphorylation of HDAC5 on two serine residues, S259 and S498. Phosphorylation of these residues induces the binding of the chaperon protein 14-3-3 with consequent activation of the nuclear export signal and export to the cytoplasm (McKinsey et al., 2001). Further, Ca^2+^ -sensitive HDAC4, which, in spite of its name lacks of known endogenous deacetylase activity, has been shown to regulate satellite cell function through promoting activation of Pax7-positive cells, although the leading mechanism remains largely unknown (Choi et al., 2014b).

High intracellular Ca^2+^ concentration also increases the activity of calcineurin serine/threonine phosphatase, which in turns dephosphorylates NFAT (Nuclear Factor of Activated T cells) transcription factors (Friday et al., 2000). Dephosphorylation unmasks a nuclear localization signal and induces NFATs nuclear translocation and gene activation (Hogan et al., 2003). Several NFAT isoforms are present in muscle cells (Hoey et al., 1995; Parsons et al., 2003; Calabria et al., 2009) and knock out studies have suggested different temporal expression patterns and function of the different isoforms (Horsley et al., 2001; Kegley et al., 2001; Horsley and Pavlath, 2002). Further, *in vitro* studies have dissected the contribution of calcineurin/NFATs in satellite cells and activated myoblasts, showing that NFATs regulate the myogenic program at least at two different steps: first, NFATc isoforms regulates *Myf5* expression in reserve cells (Friday and Pavlath, 2001), then NFATc3 cooperates with MyoD to regulate *Myogenin* gene expression in early differentiating myoblasts (Armand et al., 2008). Finally, calcineurin is also involved in fiber-type specification at the later steps of differentiation in an NFATc3 independent process (Delling et al., 2000).

### Growth Factor Activated Signaling Pathways

While CamK activity is necessary to release class II HDACs from Mef2, binding of class I HDAC to MyoD in proliferating, undifferentiated myoblasts is controlled by cell cycle dependent changes in the phosphorylation status of pRb. Dephosphorylation of Rb upon differentiation sequesters class I HDACs and allows MyoD-dependent transcription (Mal et al., 2001; Puri et al., 2001). Further, we previously showed that another family of protein kinases, namely Akt1/2, is required to recruit HATs such as p300 and CBP to the chromatin of muscle genes (Serra et al., 2007). Akt1, which is activated by IGF-1 in muscle cells, phosphorylates the C-terminal of p300 on two serines, S1734 and S1834. Phosphorylated p300 is recruited to the chromatin to ultimately lead to the hyperacetylation of histones H3 and H4 at target genes. Moreover, Akt1/2 signaling is involved in the disengagement of chromatin repressors such as Polycomb proteins upon differentiation of muscle cells (Serra et al., 2007). This is consistent with the finding that Akt1 directly phosphorylates Ezh2, the catalytic subunit of PRC2 on S21 in cancer cells, an event that modulates binding to histone H3 (Cha et al., 2005). p300 and PCAF are important not only for histone acetylation but also for the post-translational modification of tissue specific factors such as MyoD and Mef2 (Sartorelli et al., 1999; Dilworth et al., 2004; Ma et al., 2005; Serra et al., 2007). Acetylation of MyoD on residues K99, K102 and K104 is essential for recruitment to a discrete subset of genes at defined stages of the muscle program (Di Padova et al., 2007) and for proper myogenesis both *in vitro* and *in vivo* (Sartorelli et al., 1999; Duquet et al., 2006; Di Padova et al., 2007). Acetylation of Mef2, in turn, increases DNA binding and transcriptional activity (Ma et al., 2005). MyoD and Mef2 transcription factors can be also modulated by lysine methylation. In particular, it was shown that G9a methyltransferase not only increases H3K9me2 at MyoD-target genes to repress muscle gene expression (Ling et al., 2012b; Wang et al., 2013), but also directly interacts with MyoD and Mef2 to methylate them in K104 and K267 respectively, reducing their transcriptional activity (Ling et al., 2012a; Choi et al., 2014a).

In addition to IGF-1 several other growth factors have been show to signal to the chromatin in regenerating muscles. Amongst them, Hepatocyte Growth Factor (HGF) and members of the Transforming Growth Factor β (TGFβ) superfamily are fundamental in the regulation of satellite cells function.

TGFβs are potent repressors of myogenesis (Burks and Cohn, 2011). Of them, Myostatin (GDF8) is a well-known negative regulator of muscle differentiation that signals through binding to Activin type II receptors. Binding to type II receptors induces heterodimerization with type I receptors (Alk4/5) (Rebbapragada et al., 2003). Activated receptor phosphorylates Smad2/3, inducing binding to Smad4 and nuclear translocation (Moustakas, 2002). In muscle cells it has been shown that phosphorylated Smad3 binds to and interferes with MyoD and Mef2 transcriptional activity (Langley et al., 2002; Liu et al., 2004). On the contrary, Smad7, an antagonist of the canonical TGFβ/Smad signaling, promotes myogenesis through MyoD binding and activation (Zhu et al., 2004; Kollias et al., 2006). Myostatin function can be blocked by the action of Follistatin, which can be induced in muscle cells by treatment with HDAC inhibitors (HDACis; Iezzi et al., 2004). Follistatin is involved in myoblast fusion *in vitro* and contributes to the beneficial effect of HDACi treatment *in vivo* in a mouse model of muscle regeneration (Iezzi et al., 2004; Minetti et al., 2006). Further, it was recently shown that the EGF-CFC family of proteins (Shen and Schier, 2000) also antagonizes Myostatin function during muscle regeneration. In particular, Cripto, the founder member of the family, is re-expressed in activated satellite cells and positively regulates myogenesis, interfering with Myostatin-mediated Smad2 phosphorylation (Guardiola et al., 2012).

HGF is a heparin binding protein located in the extra-cellular matrix of muscle fibers. Upon injury or mechanical stretch, HGF is released from its anchor in muscle fibers by serum proteases and binds to the surface receptor c-Met, present in satellite cells (Tatsumi et al., 1998, 2002; Miyazawa, 2010). HGF has been shown to regulate satellite cell proliferation and function (Tatsumi et al., 1998; Yamada et al., 2010). Further it was recently shown that high concentrations of HGF are necessary for the re-entry of satellite cells into quiescence through a mechanism involving Myostatin up-regulation (Yamada et al., 2010). Both HGF and the inflammatory cytoquine IL-6 induce the activity of the oxidative stress sensor Nrf2 in myoblasts. Nrf2 is involved in satellite cell proliferation and repression of the myogenic program through direct down-regulation of *Myogenin* gene (Al-Sawaf et al., 2014). On the other hand, signaling through HGF/c-Met activates the mammalian target of rapamycin (mTOR) pathway in satellite cells via the PI3K/Akt cascade. mTOR is a main sensor of the metabolic status of the cell, modulating the cell response to variations in nutrient availability. Although extensive work has been done on the upstream regulators of the pathway, the downstream effectors are less known (for a detailed review on mTOR signaling, see Laplante and Sabatini, 2012). The best-characterized responses are the regulation of protein synthesis through phosphoryation of translational regulators such as eIF4E binding protein or S6 kinase and the regulation of lipid metabolism (Laplante and Sabatini, 2012). Although little endogenous mTOR is found in the nucleus, the pathway has been associated with the modulation of gene expression in skeletal muscle through the transcription factors PGC1α and YY1 (Cunningham et al., 2007; Blattler et al., 2012). A recent study showed mTOR activation upon limb-muscle injury switches quiescent satellite cells in the contro-lateral limb into what it has been called a quiescent “alert” state or G*alert*. Such state allows satellite cells to quicker activate in response to injury. Consistent with a role of the mTOR pathway in regulating the metabolic status of the cell, amongst the transcriptional changes associated to the “alert” response there is an enrichment in transcripts associated to metabolism and mitochondrial activity. The alert state is reversible and the morphological and transcriptional changes turn to those of normal quiescent satellite cells when mTOR signaling ceases. It will be interesting to dissect the molecular pathways and epigenetic changes associated to this reversible quiescence (Rodgers et al., 2014).

### Inflammatory Signals

The inflammatory infiltrate recruited to the site of lesion is the main source of secreted cytokines such as interleukina-1, interleukina-6 or Tumor Necrosis Factor (TNF) that activate muscle resident cells (Stoick-Cooper et al., 2007). In satellite cells, several signaling cascades respond to cellular stressors by converting inflammatory cues into the epigenetic information that controls gene expression (Lluís et al., 2006; Lassar, 2009). In some circumstances a single signal transducer may control multiple steps of gene regulation. One of the best studied examples of multi-layered control of gene expression by a signaling cascade is provided by the family of Mitogen Activated Protein Kinases (MAPK) α, β, γ and δ (Cuenda and Cohen, 1999; Zetser et al., 1999; Wu et al., 2000). p38 is activated in response to either inflammatory cytokines such a TNF or amphoterin/HMGB1, cell-to-cell contact and growth factors such as TGFβ in satellite cells (reviewed in Guasconi and Puri, 2009). Previous studies have shown how p38 kinases α and β contribute to the assembly of the myogenic transcriptosome on the chromatin of muscle loci, leading to the consequent activation of gene expression. p38α and β promote Mef2 transcriptional activation (Zetser et al., 1999), MyoD-E47 heterodimerization (Lluís et al., 2005) and recruitment of SWI/SNF chromatin remodeling complex (Simone et al., 2004; Serra et al., 2007; Forcales et al., 2012) and Ash2L-containing mixed-lineage leukemia (MLL) methyltransferase complex (Rampalli et al., 2007) to the chromatin of muscle genes. By contrast, activation of p38γ in satellite cells represses MyoD transcriptional activity by direct phosphorylation, which leads to the association with the H3K9 methyltransferase Suv39h1 (KMT1A) (Gillespie et al., 2009). Further, we have recently shown that p38α signaling to PRC2 represses the determination gene *Pax7* in satellite cells undergoing terminal differentiation, an event that is necessary for cell cycle exit (Palacios et al., 2010; Mozzetta et al., 2011). Activation of p38α/β downstream kinase Msk1 has been shown to regulate a chromatin switch between Ezh2-containing and Ezh1-containing PRC2 complexes at the onset of differentiation via phosphorylation of S28 on histone H3 (Stojic et al., 2011). Interestingly, Ezh1-containing complexes have been associated to polII recruitment and transcriptional activation in differentiating myoblasts, challenging the common view of PRC2 complexes as chromatin repressors (Mousavi et al., 2012).

Thus, p38 signaling can either activate or repress gene expression in satellite cells, depending on the activation status of specific p38 isoforms, or chromatin recruitment with specific modifying complexes. Furthermore, evidence supports the notion that chromatin-associated p38 kinases can control gene transcription by directly targeting components of the transcription machinery (Chow and Davis, 2006; Pokholok et al., 2006; de Nadal and Posas, 2010), suggesting a general role of p38 signaling in the control of genome redistribution of chromatin-modifying complexes in response to extrinsic signals.

Underlying a further layer of complexity, the same cytokine may modulate different intra-cellular cascades that ultimately converge to the chromatin of muscle precursors. For instance, in addition to inducing p38α/β activation, TNF also regulates NF-κB activation in muscle cells. Work from the Guttridge lab showed that the TNF/NF-κB pathway repressed *Notch* expression both in satellite cells and C2C12 myoblasts through a mechanism involving Ezh2 and Dnmt3b (Acharyya et al., 2010).

Amongst the signaling pathways activated in response to inflammatory cues in muscle, the Jun Activated Kinase (JAK)/STAT pathway responds to cytokines such as IL-6 or leukemia initiating factor (LIF). The pathway is activated upon binding of IL-6 to the IL-6R-gp130 receptor which leads to JAK activation and phosphorylation of Stat proteins on tyrosines residues. Phosphorylated Stats are able to heterodimerize and translocate into the nucleus (Stark and Darnell, 2012; Muñoz-Cánoves et al., 2013). In muscle satellite cells, nuclear Stat3 binds the regulatory regions of target genes, including *MyoD*, to regulate their expression. Consistent with a role in regulating myogenic progression (Sun et al., 2007; Wang et al., 2008), genetic depletion or pharmacological inactivation of JAK2 and/or Stat3 expanded a population of Pax7-positive, MyoD-negative cells and improved muscle regeneration (Price et al., 2014; Tierney et al., 2014).

### Developmental Programs Re-Activated in Adult Stem Cells

Several signaling pathways essential for embryonic myogenesis have been shown to be re-activated during regeneration in the adult and to play a fundamental role in the activation, commitment and differentiation of satellite cells. Here we will discuss the contribution of Notch, Wnt, Sonic Hedgehog (Shh) and Bone Morphogenetic Proteins (BMPs) pathways to adult muscle regeneration.

The Notch signaling pathway plays a fundamental role in the establishment of cell fate decisions in several tissues. In muscle it has been shown to be essential both during embryogenesis and in the adult. Activation of the Notch signaling starts with the expression of Notch ligand delta-like (Dll) in differentiating cells. Dll binds and activates the membrane receptor Notch in satellite cells, leading to the cleavage of the cytoplamic portion of the protein and nuclear translocation of the intracellular domain (NICD; Schroeter et al., 1998). NICD is a potent transcriptional activator that binds the regulatory regions of target genes together with Rbpj, the main downstream effector of the pathway (Kopan and Ilagan, 2009). It is currently widely accepted that the Notch pathway, which is activated upon injury in quiescent satellite cells, is required for maintaining the homeostasis of the satellite cells compartment and is involved in regulating proliferation and self-renewal, keeping myoblast in an undifferentiated state (Conboy and Rando, 2002; Wen et al., 2012). By using conditional knock out mice two different labs showed that Rbpj-depleted satellite cells prematurely differentiate while they fail to undergo replication, leading to premature exhaustion of the satellite cell compartment (Bjornson et al., 2012; Mourikis et al., 2012a,b). Genome-wide analysis demonstrated dynamic Rbpj chromatin binding in response to Notch activation in muscle cells, together with NICD and the acetyltransferase p300. A detailed analysis of the chromatin signature at Rbpj-bound regions, showed that different chromatin modifications are present at constitutive vs. inducible genes and confirmed the major role of Rbpj as a transcriptional activator (Castel et al., 2013). Notch signaling is switched off through ubiquitin-mediated degradation of the protein, which is directed by the cellular adaptor Numb. Numb factors comprise a family of four cytoplasmic proteins that mark Notch for proteome-mediated degradation through the E3 ubiquitin ligase Itch (McGill and McGlade, 2003). Numb has been implied in the replenishing of the satellite cell compartment by meditating asymmetric division of activated satellite cells (Shinin et al., 2006). Further, Numb-depletion has been shown to impair proliferation and differentiation of satellite cells through up-regulation of the cell cycle inhibitor p21 and Myostatin (George et al., 2013).

Work from Rando’s lab showed that a temporal switch between Notch and Wnt signaling is necessary for adult myogenesis (Brack et al., 2008). Wnt proteins comprise a family of 19 glycoproteins that play a fundamental role during embryonic myogenesis and adult homeostasis (for a detailed review on the contribution of Wnt signaling to myogenesis, see von Maltzahn et al., 2012). Secreted Wnts bind to Frizzed (Fzd) receptors, located in the plasma membrane of target cells (Sethi and Vidal-Puig, 2010). When bound to the receptor, Wnt elicits a variety of cellular responses through the activation of several intra-cellular cascades. The best known of such cascades, the canonical Wnt pathway, starts the activation of heterotrimeric G proteins and Dishevelled (Dsh) and the recruitment of Axin to the Fzd co-receptor Low Density Protein receptor (LPR)-related protein. This recruitment inactivates the βcatenin degradation complex, consisting of Axin, APC and glycogen synthase kinase 3 beta (GSK-3β). Before Wnt stimulation βcatenin levels are regulated through GSK-3β mediated phosphorylation, which targets the protein to proteasome-mediated degradation. Upon binding of Wnt ligands, stabilized βcatenin accumulates and is able to translocate into the nucleus, where it binds members of the TCF and LEF families of transcription factors. Nuclear βcatenin acts as a transcriptional co-activator (Katoh, 2007; Nusse, 2008). The canonical Wnt pathway may co-exist with other βcatenin-independent signaling, such as the activation of phospholipase C (PLC), CamkII or protein kinase C (PKC) (all of which lead to an increase in the intracellular Ca^2+^ levels) the phosphatidil-inositol 3 kinase (PI3K)/Akt/mTOR axis, the Protein Kinase A (PKA)/CREB pathway (Chen et al., 2005) and the planar-cell polarity (PCP) pathway (Kühl, 2004; Le Grand et al., 2009; von Maltzahn et al., 2011). The latest is involved in the remodeling of the cytoskeleton, acting through Fzd, Vang1, Dsh and Prickle (Montcouquiol et al., 2003; Vladar et al., 2009) Although it has not been formally proved, it was suggested the PCP pathway could modulate Carm1 (Prmt4)-dependent methylation of Pax7 in activated satellite cells (Kawabe et al., 2012). Carm1 is an arginine methyltransferase important for Pax7-mediated activation of *Myf5* in asymmetrically dividing cells through recruitment of Mll1/2 methyltransferase (McKinnell et al., 2008; Kawabe et al., 2012). It also regulates muscle-specific microRNAs, SWI/SNF-mediated chromatin remodeling and Mef2 transcriptional activity (Chen et al., 2002; Dacwag et al., 2009; Mallappa et al., 2011).

The role of the Wnt proteins in adult muscle regeneration is complex as illustrated by the amount of family members whose expression is modulated during regeneration (Polesskaya et al., 2003; Brack et al., 2008). In regenerating muscle Wnts are released by the myofibers (Polesskaya et al., 2003) whereas the Fzd receptor is activated only in satellite cells (Brack et al., 2008). Numerous studies have helped to clarify the multi-step control of myogenesis through sequential activation of different Wnt ligands and pathways. First, Wnt7-activation of the PCP pathway stimulates symmetric expansion of satellite cells (Le Grand et al., 2009). Then, activation of the canonical Wnt/βcatenin pathway was proposed to be required for the differentiation of progenitor cells (Polesskaya et al., 2003; Rochat et al., 2004; van der Velden et al., 2006; Brack et al., 2008). Consistently, genetic depletion of Bcl9, the mammalian homologue of the Wnt co-regulator in drosophila *legless*, in Pax7-positive cells abrogates nuclear localization of βcatenin and impairs injury-mediated muscle regeneration (Brack et al., 2009). However, a recent study has challenged this idea, showing that although the Wnt pathway is activated during regeneration, this activation is transient and it is the subsequent Wnt inactivation, rather than activation, that is necessary for proper regeneration (Murphy et al., 2014). Finally, it has been shown that, upon injury, Wnt signaling is also able to induce *Pax7* expression in a subpopulation of muscle-resident CD45+ stem cells through a canonical βcatenin pathway (Polesskaya et al., 2003). However, the contribution of this population of CD45+ cells to normal adult myogenesis is controversial and it is probably limited to pathological conditions such as muscular dystrophies, acting as a compensatory mechanism to the continuous waves of degeneration ad regeneration that lead to the exhaustion of the satellite cells compartment.

BMPs are members of the TGFβ super-family. As with the canonical TGFβ pathway binding of BMPs to type I and II receptors induces phosphorylation of Smad 1, 5 and 8. Phosphorylated Smad1/5/8 interact with Smad4 to form an heterodimer that translocates to the nucleus and binds the chromatin of target genes (Canalis et al., 2003). Upon muscle injury, type IA BMP receptor is activated in satellite cells, and Smad proteins are phosphorylated. Activation of the pathway induces satellite cells proliferation while blocking differentiation (Ono et al., 2011). At the onset of differentiation, the increase of BMP antagonists such as Noggins or Chordin antagonizes BMP signaling to allow satellite cells differentiation (Friedrichs et al., 2011; Ono et al., 2011). Further, it was previously shown that Notch signaling is necessary for BMP-induced block of differentiation, highlighting the functional interplay amongst the two pathways (Dahlqvist et al., 2003).

Hedgehog (hh) is an evolutionary conserved pathway essential for tissue morphogenesis during development. The pathway is activated upon interaction of the extra-cellular ligands Shh, Ihh and Dhh with the transmembrane receptor Patched1 (Ptch1), which leads to the activation of smoothened (Smo) and translocation to the nucleus of Gli transcription factors (Jiang and Hui, 2008). Of the developmental programs that are reactivated upon muscle injury the role of Shh as a regulator of stem cell function in adult muscle has been by far the less studied. It was recently shown that the primary cilia activate Shh at the initial stages of muscle differentiation and is essential for proper differentiation (Fu et al., 2014). Shh activation induces proliferation of *ex vivo* cultured mouse satellite cells while inhibiting terminal differentiation. In addition, upon induction of differentiation, Shh regulates caspase-3 activation and apoptosis (Koleva et al., 2005). On the contrary, a different study using primary cultures of chicken myoblasts points out to a role of the signaling pathway both in myoblasts proliferation and differentiation through the MAPK/ERK and Akt1 pathways (Elia et al., 2007). Recently it was shown Shh is reactivated during muscle injury *in vivo* and pharmacological inactivation of the pathway reduces the number of myogenic progenitors at the site of lesion and impairs regeneration (Straface et al., 2009). Despite the fact little is known on how Shh modulates chromatin structure in adult muscle cells, it was previously shown that Gli2, together with Zic1 and Pax3, activates *Myf5* epaxial enhancer during somitogenesis (Himeda et al., 2013). Consistent with a role in regulating master regulatory factors, works on developing limbs have mapped genome-wide Gli3 binding by ChIP-seq, showing Shh-responsive genes are associated to gene categories such as development and morphogenesis and are enriched in transcriptional regulators (Vokes et al., 2008; Shi et al., 2014). Further, a recent study has shown that Shh induces an epigenetic switch consisting on disengagement of PRC2 and recruitment of the H3K27me3 demethylase Jmjd3 at target loci in responsive fibroblasts, leading to gene activation (Shi et al., 2014). If similar mechanisms are active also in satellite cells still needs to be elucidated.

### Redox Status

Upon injury, inflammatory cytokines can alter the physiological redox status of skeletal muscle stem cells, a fundamental prerequisite for muscle regeneration. Important players in redox status determination are Nitric Oxide (NO), Reactive Oxygen Species (ROS) and the NAD^+^/NADH ratio.

NO is a free radical synthesized from the amino acid L-arginine by three different isoforms of NO synthase (NOS; Nathan and Xie, 1994). In skeletal muscle NO is produced constitutively by neuronal type NO isoform (nNOS; Nakane et al., 1993; Silvagno et al., 1996) that controls its physiological production to hinder a potential toxicity. NO has been described as an epigenetic molecule (Colussi et al., 2008; Nott et al., 2008) capable of inducing global epigenetic modification in Duchenne muscular dystrophy (DMD; Colussi et al., 2009). In DMD, the absence of dystrophin causes delocalization of the dystrophin-associated complex (DAPC) from the cytoskeleton of muscle fibers membrane, leading to structural destabilization of the sarcolemma (Matsumura et al., 1994; Ervasti and Sonnemann, 2008). DAPC displacement determines the dissociation of its interactor, the sarcolemma neuronal NO synthase (nNOS) from the same site, causing an alteration in NO production (Brenman et al., 1995). In healthy muscle, NO deposits S-nytrosilation on HDAC2, which is released from the chromatin, an event that activates a specific pattern of gene expression, including the activation of several microRNAs. Conversely, in dystrophic muscles a decrease of nNOS activity reduces HDAC2 S-nytrosilation, increasing chromatin binding and determining the repression of target genes and microRNAs. Amongst these, miR-1 repression up-regulates G6PD levels, which sensitizes muscle cells to physiological production of free radicals. This contributes to high oxidative stress levels observed in DMD (Cacchiarelli et al., 2010). When dystrophin is rescued by exon skipping, the recovery of correct nNOS localization on the membrane induces chromatin disengagement of HDCA2 and re-expression of its target genes, contributing to late muscle differentiation (Cazzella et al., 2012).

Differently from NOS, ROS production occurs at different locations in muscle fiber including the sarcoplasmic reticulum, transverse tubules, sarcolemma and the cytosol but the main sites are the mithocondria (Barja, 1999). Changes in redox status of muscle fibers modify kinases and phosphatases activities causing alterations in gene expression (Chiarugi and Cirri, 2003; Torres and Forman, 2003). For example p38 MAPK and JNK are activated not only in response to inflammatory cytoquines but also in response to ROS production (Cuschieri and Maier, 2005) Increases in ROS concentration induces a strong depletion of the glutathione (GSH) pool that leads to NF-κB activation and reduction of *MyoD* expression, impairing myogenesis (Guttridge et al., 1999; Ardite et al., 2004). NF-κB is known for its negative regulation of skeletal muscle differentiation (Buck and Chojkier, 1996; Langen et al., 2001) even though in response to ROS alteration is also able to promote the activity of inducible NOS whose role in muscle differentiation is controversial (Kaliman et al., 1999; Piao et al., 2005). Another protein, p66Shc, an isoform of Src homology 2 domain containing transforming protein 1 (Shc), is phosphorylated in response to elevated ROS levels, negatively contributing to myogenesis. p66Shc KO mice show higher regenerative capacity and differentiation of skeletal muscle stem cells compared to wt mice. Probably, active p66Shc produces superoxide anions, which deplete available NO by forming peroxynitrite, which is not generated in KO mice (Zaccagnini et al., 2007). Moreover at high ROS concentration, the ratio of NAD^+^/NADH is shifted in favor of NAD^+^, which promotes the activity of a family of NAD^+^-dependent HDACs, the sirtuins. Sirt1 (the mammalian homologue of yeast Sirtuin2 (Sir2))—mediates MyoD deacetylation and inhibition of transcription (Fulco et al., 2003). Sirt1 senses variations in NAD^+^/NADH ratio during skeletal muscle cell differentiation (MacDonald and Marshall, 2000). In undifferentiated cells, where this ratio is high, MyoD is kept inactive by Sirt1 mediated hypoacetylation whereas when such ratio decreases, skeletal muscle cells start to differentiate. Recently Abdel-Khalek et al. ascribed to Sirt3, a mitochondrial NAD^+^ dependent deacetylase, a role in the regulation of myoblast differentiation (Abdel Khalek et al., 2014). Differently from Sirt1 that is highly expressed in proliferating myoblasts, Sirt3 expression starts to increase when C2C12 cells arrive at confluence and its levels are kept elevated during differentiation. Interestingly, Sirt3-depleted cells show a block of differentiation, high levels of ROS, a decrease in manganese superoxide dismutase (MnSOD) activity and an inhibition of Sirt1 expression (Abdel Khalek et al., 2014). It would be interesting to clarify the mechanism by which Sirt3 regulates *Myogenin* and *MyoD* expression and why Sirt1 is not up-regulated upon Sirt3 depletion.

All together, these data demonstrate that the maintenance of cellular redox homeostasis, as a result of a crosstalk among different free radicals, represents another layer for regulating muscle differentiation through chromatin signaling.

### Mechano-Transduction

Due to their particular anatomical position, muscle stem cells are strongly exposed to physical and mechanical cues such as contraction and/or changes in the extra-cellular matrix stiffness (Dupont et al., 2011; Gilbert et al., 2011). Cells convert these mechano-stimuli into biochemical and nuclear signals through the Yap and Taz mediators (Dupont et al., 2011) that usually control cell growth and differentiation. Yap and Taz are the nuclear transducers of the Hippo pathway (Pan, 2010).

The Hippo pathway is a signal transduction pathway involved in development, cell function, regeneration and organ size in many tissues and is altered in several human diseases including muscular dystrophy and cancer (Tremblay and Camargo, 2012; Yu and Guan, 2013; Tremblay et al., 2014; Wackerhage et al., 2014). The central Hippo cascade comprises upstream elements such as the STE20-like protein kinases 1 and 2 (Mst1 and Mst2) and the large tumor suppressor kinases 1 and 2 (Lats1 and Lats2). Phosphorylation and subsequent activation of Lats1/2 by Mst1/2 induces Yap/Taz phosphorylation (Huang et al., 2005) Phosphorylation of the best-characterized phospho-sites on Yap (S127) and on its analogous Taz (S89) determines their retention and inactivation in the cytosol by 14-3-3 proteins (Basu et al., 2003; Wackerhage et al., 2014). In response to different stimuli such as G-protein coupled Receptor (GPCR) signaling (Yu et al., 2012), mechano-stimuli (Dupont et al., 2011) and apico-basal polarity (Huang et al., 2005) Yap and Taz are dephosporylated and translocate to the nucleus where they are recruited to the chromatin through binding to several transcription factors (Hong and Guan, 2012).

In muscle cells Taz and Yap associate with TEAD transcription factors (TEA domain), which bind to MCAT elements (muscle C, A and T; 5′-CATTCC-3′) located in the promoter or enhancer regions of key genes that regulate commitment (*MyoD, Myf5, Mrf4*), proliferation (*Cyclin D1*) and differentiation (*Myogenin*) of satellite cells. Both Yap and Taz are expressed in skeletal muscle (Jeong et al., 2010; Watt et al., 2010). The group of Wackerhage was the first to assess the expression of Yap in skeletal muscle cells. In particular they have shown that in myoblasts, unphoshorylated Yap is localized in the nucleus where positively controls the expression of proliferation genes. Conversely, as C2C12 undergo differentiation, Yap (S127) phosphorylation increases and the protein translocates from the nucleus to the cytosol where it is kept inactive (Watt et al., 2010). Moreover Yap stimulates proliferation of activated satellite cells and prevents their differentiation by controlling expression of genes associated with cell cycle, ribosome biogenesis and modulation of myogenic differentiation (Judson et al., 2012). In contrast to what observed for Yap, ectopic expression of *Taz* promotes myogenic differentiation through a cooperative interaction with MyoD that increases its binding to DNA (Jeong et al., 2010). Taz has been shown to interact also with Pax3 during development (Murakami et al., 2006). Jeong et al. demonstrated Taz levels increase after muscle injury in mice suggesting it is also involved in muscle regeneration (Jeong et al., 2010). Under differentiation conditions, translocation of Taz to the nucleus is required for enhancing the expression of genes such as *Myogenin, Mhc and Mck*. This process can be stimulated by selenoproteinW which induces Taz nuclear translocation by interrupting its binding with 14-3-3 protein and consequently increasing myogenic differentiation (Jeon et al., 2014). Recently, compounds that promote myogenesis through Taz activation in C2C12 cells have been characterized and shown to induce an increase in *Taz* expression, interaction with MyoD and MyoD recruitment to the chromatin during the initial phase of the muscle differentiation program (Park et al., 2014). Collectively these data point out to Yap and Taz as novel components of the muscle transcriptosome and suggest they could be potential therapeutic targets in muscular dystrophies. Their opposite effects on proliferation and differentiation suggest that fine-tuned modulation of Taz and Yap activity might be a good strategy for increasing muscle regeneration and amelioration of diseased phenotypes.

**Figure 1:**
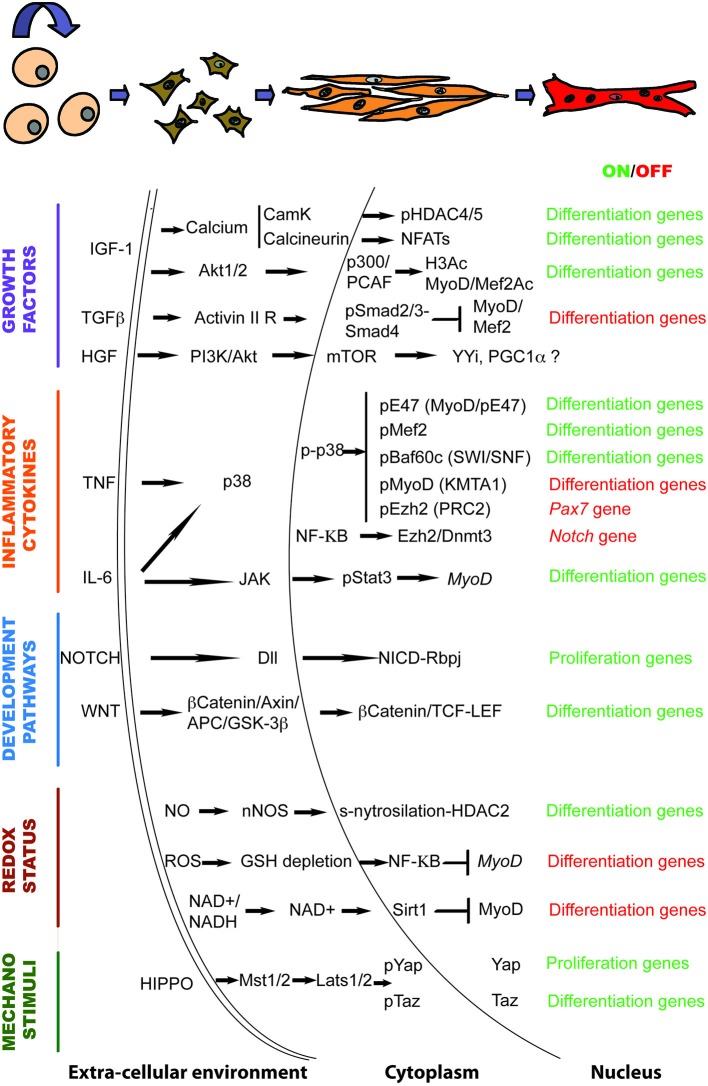
**The genetic and epigenetic signaling governing muscle regeneration**. Schematic representation of the signaling pathways and downstream chromatin effectors that regulate the transition from activated satellite cells to multinucleated myotubes in response to regeneration cues.

Altogether, the data discussed here suggest that signaling to the chromatin in response to the regenerative microenvironment plays a crucial role during muscle regeneration. They are summarized in Figure 1.

## Age-Dependent Decline of Satellite Cell Function

Aging is associated to a loss of the homeostatic and regenerative potential in all tissues and organs. In muscle, it is reflected on a loss of muscle mass and strength (sarcopenia) and to an impaired regeneration potential associated to a dysfunction of the satellite cell compartment (Brack and Rando, 2007).

Both cell-intrinsic changes and changes within the regenerative microenvironment are associated with the stem cell loss of function that occur with age. For instance, replicative damage, proteomic changes (i.e., accumulation of damaged macromolecules) and changes in the epigenome and the proteome have been shown to affect stem cell function in old tissues (Liu and Rando, 2011; García-Prat et al., 2013; Sinha et al., 2014). Amongst the cell-intrinsic changes, modification of the epigenetic profile, including changes in the DNA methylation patterns and post-translational histone modifications are particularly interesting, as they are potentially reversible. Recent work has shown a progressive increase in DNA methylation in aging muscle (Day et al., 2013; Jin et al., 2014; Ong and Holbrook, 2014; Zykovich et al., 2014). Interestingly, key studies in tumor and aged cells indicate that *de novo* DNA methylation in the adult is usually restrained to a subset of CpG islands, most of which are PRC2 target regions (Schlesinger et al., 2007; Bergman and Cedar, 2013). Consistent with this, satellite cells isolated from old mice show altered levels and distribution of H3K27me3 (Liu et al., 2013). The functional interplay between PRC2 and the DNA methylation machinery in aged satellite cells likely contributes to the impaired regeneration potential of old muscles and could represent novel pharmacological target to improve muscle function in aged individuals.

On the other hand, elegant parabiotic studies showed that changes in the regenerative environment of old muscles regulate satellite cell function (Conboy et al., 2005; Brack et al., 2007; Sinha et al., 2014). For instance, attenuated Notch signaling leads to reduced satellite cells proliferation and impaired regeneration in aged muscle. Muscle regeneration can be recovered by ectopic activation of Notch or by exposing old animals to a young environment (Conboy et al., 2005). On the contrary, a progressive increase in Wnt signaling with age alters muscle stem cell fate (Brack et al., 2007; Doi et al., 2014), inducing a myo-to-fibrogenic conversion of muscle progenitors that leads to increased fibrosis *in vivo* (Brack et al., 2007). As in the case of impaired Notch signaling, the fibro-adipogenic conversion observed in old muscles is reversible and can be reduced by exposing mice to conditioned serum from young animals. Conversely, *in vivo* activation of the pathway in young mice by injection of Wnt3A increases fibrotic deposition (Brack et al., 2007). Finally, it was recently shown that the TGFβ member Growth Differentiation Factor 11 (GDF11) also reverses the age-related changes in satellite cells dysfunction (Sinha et al., 2014).

The contribution of an old inflammatory infiltrate to the impaired regenerative response of aged muscles can be further predicted by the elevated levels of cytokines such as IL-1, IL-6 and TNF observed in old muscles (Phillips and Leeuwenburgh, 2005; Dirks and Leeuwenburgh, 2006). In a recent study Trendelenburg et al. showed that elevated levels of IL-1 and TNF block differentiation of human myoblasts through activation of a signaling pathway involving TGFβ activated kinase (TAK1)/p38/NF-κB and leading to increased expression of ActivinA (Trendelenburg et al., 2012). Increased levels of TNF, together with alterations in the pathway of FGFR1, also lead to aberrant activation of the p38α/β cascade in satellite cells derived from old muscles. Two recent studies have highlighted the importance of p38α/β in maintaining satellite cells function and homeostasis during their lifespan, showing that increased p38α/β activity leads to a cell-autonomous defect in satellite cells self-renewal and activates a senescence program. This p38α/β-dependent decline in satellite cells function with age cannot be overcome by exposure to a young microenvironment but instead is partially reversed when satellite cells are treated with the p38α/β inhibitor SB203580 (Bernet et al., 2014; Cosgrove et al., 2014). Although in these experiments p38α/β blockade was performed *ex vivo* prior to satellite cell transplantation, pharmacological manipulation of the p38α/β signaling has been achieved also *in vivo* through the use of antibodies against TNF receptor (Palacios et al., 2010). Acute treatment with neutralizing TNF antibodies expands a population of Pax7+, differentiation-competent satellite cells in young animals (Palacios et al., 2010), whereas long-term treatment with the same antibodies have been shown to have a beneficial effect on muscle regeneration (Radley et al., 2008; Huang et al., 2009). If such beneficial effects are also obtained in old mice needs to be investigated. Altogether these studies highlight the potential impact of pharmacological approaches in recovering satellite cell function in diseased and aged muscles.

*In vivo* pharmacological manipulation of a discrete chromatin signaling has in fact been the strategy adopted by the groups of Rudnicki and Sacco to stimulate the function of old satellite cells. In this case, interfering with an altered IL6/JAK/Stat3 signaling through the use of JAK/STAT inhibitors increases the regeneration potential of old and dystrophic muscles (Price et al., 2014; Tierney et al., 2014), revealing its potential for pro-regenerative therapy to counteract the impaired regeneration observed with age.

Even if it is becoming increasingly clear that satellite cells malfunction is highly modulated by age-related changes in the surrounding microenvironment, a gap of knowledge persists on how alterations in the old regenerative environment and cell-intrinsic changes (i.e., changes in the metabolism or in the epigenetic profile) converge to modulate the function of aged satellite cells. The relative contribution of aberrant environmental cues and cell-intrinsic changes to aged satellite cells function can now be partially explained by work in Muñoz-Cánoves lab. By distinguishing old (20–24 months) from geriatric (over 28 months) mice, Sousa-Victor et al. have dissected the transcriptional changes associated to the aging process, that ultimately depends on Polycomb-mediated silencing of the p16/INK4 locus (Sousa-Victor et al., 2014). These data provide a partial explanation of why pre-senescent satellite cells are responsive to a young environment whereas full gero-conversion is an irreversible, cell autonomous, process. Future studies aimed to investigate in detail the signals and molecular mechanisms driving to this point of no return will be fundamental for the development of novel therapeutic approaches to delay muscle miss-function with age.

Remarkably, in a recent article from this special issue of Frontiers in Aging Neurosciences, Formicola et al. demonstrate that, contrary to what happens in limb muscles, the extra-ocular muscles (EOM) stem cell niche is resistant to age and disease (Formicola et al., 2014), probably thanks to the contribution of a population of muscle-resident cells called PICs (PW1 interstitial cells) (Mitchell et al., 2010; Pannérec et al., 2013). If this resistance to aging is due to a direct contribution of PICs to muscle regeneration or to indirect modulation of satellite cells function still needs to be investigated. It will also be interesting to understand what makes EOM PICs and maybe other EOM muscle-resident cell populations such FAPs (Fibroadipogenic precursors) more resistant to age-dependent cell-intrinsic changes. FAPs are multi-potent mesenchymal cells located in the interstitium of muscle fibers (Joe et al., 2010; Uezumi et al., 2010). During muscle regeneration they support the myogenic potential of muscle stem cells (Uezumi et al., 2010; Mozzetta et al., 2013) through the release of paracrine factors such as Follistatin, the functional antagonist of Myostatin (Mozzetta et al., 2013). In DMD at late stages of the disease, the continuous waves of degeneration and regeneration leads to the conversion of FAPS into fibro-adipocytes, responsible of the fat and fibrotic deposition that characterize DMD muscles (Joe et al., 2010; Uezumi et al., 2010; Mozzetta et al., 2013). It has been recently shown that treatment with HDACi increases the regeneration potential and prevents the fibro-adipogenic degeneration of young, but not old, dystrophic muscles (Minetti et al., 2006; Consalvi et al., 2013; Mozzetta et al., 2013). HDACis in young mice act in part by regulating the fate of FAPS towards the myogenic lineage, through a mechanism involving a microRNA-dependent control of SWI/SNF subunit composition (Saccone et al., 2014). On the contrary, FAPs from old dystrophic muscles are resistant to HDACi-induced chromatin remodeling at muscle loci and fail to activate the pro-myogenic phenotype (Saccone et al., 2014). Therefore, age-dependent cell-intrinsic changes in other muscle-resident populations can also alter the regeneration potential of the satellite cell pool.

## Conclusions and Perspectives

Muscle regeneration is a multi-step process that entails the coordinated activation and repression of discrete transcriptional programs. In this review we aimed to highlight the key role of signaling pathways in transmitting the extra-cellular information to the chromatin of satellite cells to regulate gene expression. The temporal pattern of activation in response to extra-cellular cues, the interplay amongst the pathways and the activation and/or repression of downstream effectors are fundamental for the fine-tuned control of muscle regeneration. Deciphering the muscle-specific chromatin signaling is particularly important for the rational design of novel pharmacological approaches aimed to improve satellite cells function in old and diseased muscles. Being pharmacologically manipulable and potentially reversible, both signaling cascades and the epigenome are attractive targets for the design of novel therapeutic interventions.

Finally, numerous studies have revealed a striking similarity between adult and embryonic myogenesis, regarding the pathways and the mechanisms affecting muscle stem cell function (see Buckingham and Rigby, 2014). Here we have focused on how adult muscle stem (satellite) cells activate the differentiation program in response to regeneration stimuli. Despite less studied, we speculate similar mechanisms are also active during embryonic myogenesis and participate to the successful completion of the myogenic program in response to developmental cues.

## Conflict of Interest Statement

The authors declare that the research was conducted in the absence of any commercial or financial relationships that could be construed as a potential conflict of interest.
